# Bloodletting therapy for treating patients with chronic urticaria: A systematic review and meta-analysis protocol

**DOI:** 10.1097/MD.0000000000014541

**Published:** 2019-02-15

**Authors:** Qin Yao, Xinyue Zhang, Yunnong Mu, Yajie Liu, Yu An, Baixiao Zhao

**Affiliations:** Beijing University of Chinese Medicine, Beijing, China.

**Keywords:** bloodletting therapy, chronic urticaria, complementary medicine, protocol, systematic review

## Abstract

**Background:**

Chronic urticaria is a common disease affecting patients’ quality of life, and leading to substantial burden to both patients and society. Many trials have shown that bloodletting therapy is effective in treating chronic urticaria. There are currently no systematic reviews of bloodletting therapy for chronic urticaria. This protocol aims to present the methods used to assess the effectiveness and safety of bloodletting therapy for patients with chronic urticaria.

**Methods:**

The following databases will be searched from their inception: the Cochrane Central Register of Controlled Trials (CENTRAL), PubMed, EMBASE, China National Knowledge Infrastructure (CNKI), Chinese Biomedical Literature Database (CBM), Chinese Scientific Journal Database (VIP database), and Wan-Fang Database. Clinical randomised controlled trials related to bloodletting therapy for treating chronic urticaria will be included, regardless of publication status and languages. Study selection, data collection, and quality assessment will be independently conducted by 2 researchers. For data synthesis, we will select either the fixed-effects or random-effects model according to heterogeneity assessment. Disease activity control will be assessed as the primary outcomes. Response rate, recurrence rate and adverse events will be evaluated as secondary outcomes. If it is appropriate for meta-analysis, RevMan V.5.3 statistical software will be used. Otherwise, a systematic narrative synthesis will be conducted. The results will be presented as risk ratio (RR) with 95% confidence intervals (CIs) for dichotomous data and weight mean difference (WMD) or standard mean difference (SMD) 95% CIs for continuous data.

**Dissemination and ethics:**

The protocol of this systematic review will be disseminated in a peer-reviewed journal and presented at relevant conferences. It is not necessary for a formal ethical approval because the data are not individualised.

**Trial registration number:**

PROSPERO CRD42018111143.

## 1 Introduction

### 1.1 Description of the condition

Chronic urticaria (CU) is a common disease characterized by the development of wheals, angioedema or both, with a duration of more than 6 weeks.^[1]^ The prevalence of CU ranges 0.5% to 1% and approximately 1 in 5 individuals suffers from urticaria at least once during their lifetime.^[2]^ Urticaria occurs in all age groups, while a higher incidence is displayed in individuals between 20 and 40.^[2]^ CU is a debilitating disease, which impairs patients’ quality of life and affects their performance at work and school.^[1–3]^ Both patients’ objective functioning and subjective well-being are being affected by CU.^[4–6]^ CU also lead to substantial economic burden to patients and society owing to its high medication costs.^[7]^

Depending on the underlying causes, CU can be divided into chronic spontaneous urticaria (CSU) and chronic inducible urticaria (CIndU), with a ratio of almost 2:1.^[1,8]^ CIndU consists of several subtypes, including symptomatic dermographism, cold urticaria, delayed pressure urticaria, solar urticaria, heat urticaria, vibratory angioedema, cholinergic urticaria, contact urticarial, aquagenic urticaria.^[1]^ The EAACI/GA^2^LEN/EDF/WAO guideline has recommended a diagnostic work up for chronic urticaria.^[1]^ To diagnose CSU, a thorough history, comprehensive physical examination and further appropriate diagnostic tests should be performed to exclude differential diagnoses and identify triggers of exacerbation or underlying causes. Special instruments, including urticaria activity score (UAS), the angioedema activity score (AAS), the CU quality of life questionnaire (CU-Q2oL), the angioedema quality of life questionnaire (AE-QoL) and the urticaria control test (UCT) should be assessed for disease activity, impact and control.^[9–11]^ To diagnose CIndU, it is important to identify the subtype of CIndU and to determine trigger thresholds.^[10]^

The management of CU is aimed at complete symptom control,^[1]^ including the following therapeutic approaches: the identification and elimination of underlying causes, the avoidance of eliciting factors, tolerance induction, and/or the use of pharmacological treatment.^[1]^ Modern 2nd-generation H_1_-antihistamines is recommended as the first-line pharmacological treatment.

### 1.2 Description of the intervention

Bloodletting therapy (BLT), also known as phlebotomy, blood donation, or collateral pricking therapy, is an intervention to treat diseases through the removal of a small amounts of blood from patients.^[12]^ BLT has been widely used around the world for many centuries.^[13–15]^ In the West, BLT has long been one of the main therapies since the time of Hippocrates.^[14]^ In China, BLT originated from primitive society and was usually used for patients who have excessive, heat or stasis syndromes.^[16]^ Besides, the instruments, locations, methods and volume of BLT varies in different diseases and cultures.^[17,18]^

Nowadays, BLT is still widely used to treat a slew of totally different diseases, for example, fever, infections, bronchopneumonia, hemochromatosis, porphyria cutanea tarda, polycythemia vera, diabetes, insulin-resistant iron overload syndrome, and iron accumulation hypothesis.^[19]^ A large number of clinical trials have reported that BLT is effective for the treatment of urticaria.^[20–22]^

### 1.3 How the intervention might work

In TCM theory, it is speculated that the potential mechanism of bloodletting therapy is re-harmonizing the balance of Qi-Blood circulation, dredging the channels and collaterals, and draining the body's heat or excess energy.^[23–25]^ In modern medicine, the mechanism of bloodletting therapy is yet not clear.

### 1.4 Why it is important to do this review

Bloodletting therapy stemmed from thousands of years ago and it has been extensively used in dermatosis.^[24,26,27]^ Numerous studies have reported the effect of bloodletting therapy in treating chronic urticaria in China.^[28–30]^ So far, there has had no available systematic review evaluating its effectiveness and safety. Therefore, it is necessary for us to investigate the evidence of bloodletting therapy for chronic urticaria. In this article, we present the protocol of our proposed systematic review in bloodletting therapy for chronic urticaria patients.

### 1.5 Objectives

To systematically evaluate the effectiveness and safety of bloodletting therapy for patients with chronic urticaria.

## 2 Methods and analysis

This protocol has been drafted under the guidance of the Preferred Reporting Items for Systematic Reviews and Meta-analysis Protocols (PRISMA-P) and Cochrane handbook for systematic reviews of interventions.^[31,32]^ This systematic review will be conducted between 29 September 2018 and 31 March 2019. Before started, a consistency training will be carried out to ensure that all reviewers have a basic understanding of the background, the purpose and the process of the review.

### 2.1 Inclusion criteria for study selection

#### 2.1.1 Types of studies

Randomized controlled trials (RCTs) will be included, without restrictions on language and publication status. Randomized crossover studies and quasi-randomised trials will be excluded.

#### 2.1.2 Types of participants

Patients with chronic urticaria, regardless of sex, age, race, or educational and economic status. Trials used with validated diagnose criteria will be included, for examples, EAACI/GA2LEN/EDF/WAO guideline, Chinese guidelines for the diagnosis and treatment of urticarial.^[1,33–38]^

#### 2.1.3 Types of interventions and comparisons

Bloodletting therapy is defined as the practice of letting blood out to cure a patient by sharp instruments, with or without an auxiliary method like cupping or leeches.^[14]^ The sharp instruments includes triangle-edged needle, plum blossom needle, injection needle, dermal needle, blades, vacuum blood sampling needle etc.^[39–41]^ Bloodletting therapy combined with a different type of complementary therapy (e.g., Chinese herb decoction, acupuncture and other therapies) will be excluded. The following treatment comparisons will be investigated:

1. Bloodletting therapy compared with no treatment.
2. Bloodletting therapy compared with placebo or sham Bloodletting therapy.
3. Bloodletting therapy compared with other active therapies.
4. Bloodletting therapy in addition to active therapy compared with the same active therapy.

### 2.2 Types of outcome measures

#### 2.2.1 Primary outcomes

Disease activity control will be assessed through the primary outcome, using urticaria activity score (UAS), urticaria control test (UCT) or other validated symptom scores.^[1]^

#### 2.2.2 Secondary outcomes

The following aspects will be assessed as the secondary outcomes:

1. Response rate.
2. Quality of life, using chronic urticaria quality of life questionnaire (CU-Q2oL) etc.
3. Recurrence rate during the follow-up period.
4. Adverse events.

### 2.3 Search methods for identification of studies

The following databases will be searched from their inception to November 2018, by 2 independent review authors (YM and YL): the Cochrane Central Register of Controlled Trials (CENTRAL); PubMed; EMBASE; the Web of Science; Traditional Chinese Medicine databases; China National Knowledge Infrastructure (CNKI); Chinese Biomedical Literature Database (CBM); Chinese Scientific Journal Database (VIP database); and Wan-Fang Database. The publication status and languages will not be restricted. One more retrieval will be conducted by the 2 researchers (YM and YL) to ensure the latest studies could be included before this review is completed. A search strategy for Medline database has been established on the guidance of the Cochrane handbook guidelines (Table 1).^[42]^ Similar search strategies will be conducted in all the other databases.

**Table 1:**
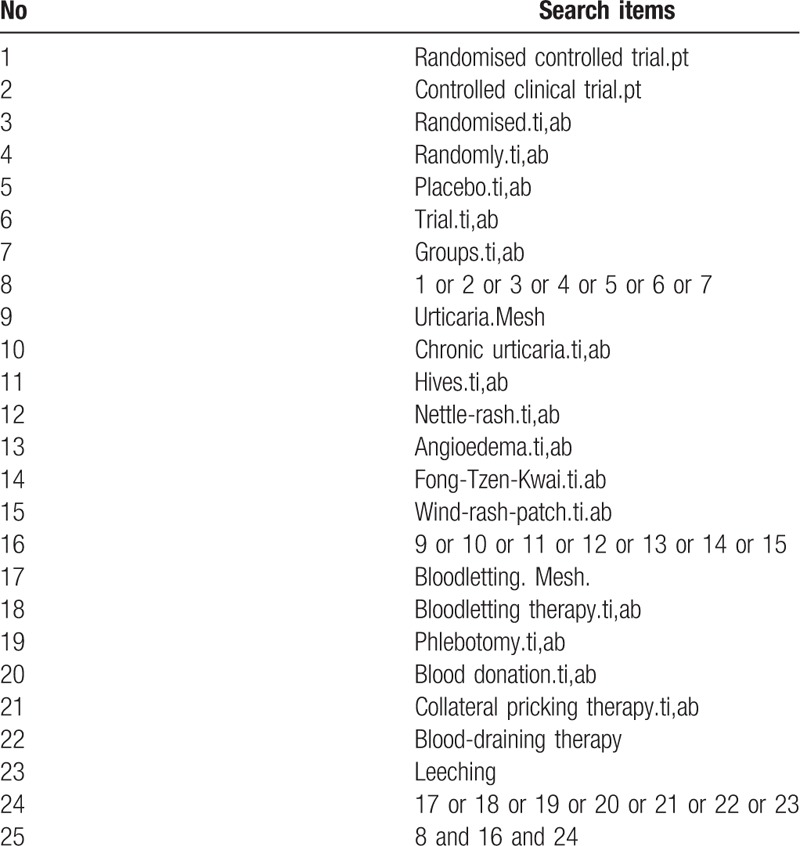
Search strategy used in PubMed.

Additionally, we will search the reference lists of included studies and published reviews related to bloodletting therapy and acupuncture for potential eligible studies. We will also search the conference abstracts and trial registered platforms to obtain ongoing or unpublished trials. The following trial registered platforms will be searched: Clinicaltrials.gov (http://www.clinicaltrials.gov) and the World Health Organization International clinical trials registry search portal (http://apps.who.int/trialsearch/).

### 2.4 Data collection and analysis

#### 2.4.1 Selection of studies

**Figure 1:**
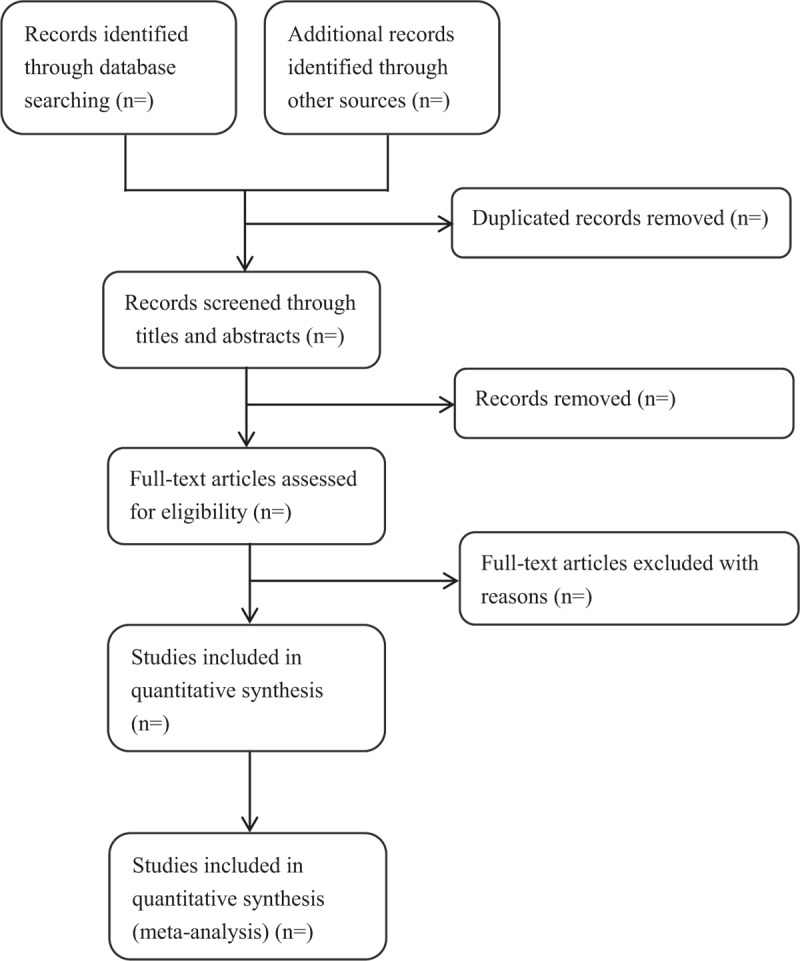
Flow diagram of the study selection process.

EndNote software (V.X7) software will be used to remove duplicates and manage the trials that have been searched. Two review authors (YM and YL) will independently review and screen the titles and abstracts of all retrieved studies to confirm eligible trials. The full text will be scanned if the studies cannot be identified after the screening of titles and abstracts. Excluded studies will be recorded with the reasons of their exclusion. In this process, disagreements will be discussed by the 2 authors (YM and YL) and be arbitrated by the third author (BZ) when the 2 authors cannot reach consensus. The authors of the original studies will also be contacted for clarification when necessary. The process of the selection is shown in Figure 1.

#### 2.4.2 Data extraction and management

Before extraction, a consistency assessment will be performed between the 2 authors (YA and XZ). They will respectively extract data in a small scope of trials using a predefined extraction form, which will be designed by all of the reviewers. After a common consensus is reached, they will independently collect data and fill in the data extraction form from included trials for the following information: general information, participants, methods, interventions, outcomes, results, adverse events, conflicts of interest, ethical approval, and other information. In this process, any disagreement will be discussed between the 2 authors, and further disagreements will be judged by the third author (BZ).

#### 2.4.3 Assessment of risk of bias in included studies

The Cochrane Collaboration's tool for risk of bias assessment will be used for the evaluation of methodological quality. Two authors (YA and XZ) will conducted the assessment independently for all included studies. The following domains for risk of bias will be assessed: sequence generation, allocation sequence concealment, blinding of participants and personnel and outcome assessors, incomplete outcome data, selective outcome reporting, and other sources of bias. The assessments will be classified into 3 levels: low risk, high risk, and unclear risk. Any disagreements will be discussed and arbitrated by the third author (BZ).

#### 2.4.4 Measures of treatment effect

For continuous data, weight mean difference (WMD) or standard mean difference (SMD) with 95% confidence intervals (CIs) will be applied to measure the treatment effect. For dichotomous data, risk ratio (RR) with 95% CIs will be applied to measure the treatment effect.

#### 2.4.5 Unit of analysis issues

Data that are from studies with parallel-group will be selected for analysis. In cross over trials, only the first phase data will be considered. In trials with multiple observation nodes, only data at the end of the treatment or the end of the follow-up will be extracted for assessment. In all studies, a single measurement for each outcome from each participant is collected and analysed.

#### 2.4.6 Dealing with missing data

We will do our utmost to contact the first or corresponding authors of the included studies to get missing or insufficient trial data by email or telephone. If the additional data cannot be obtained, only the available data will be analyzed, followed by a discussion to judge the potential impact of the missing data.

#### 2.4.7 Assessment of heterogeneity

Higgins I^2^ statistic will be used to quantify heterogeneity among the included studies.^[32]^ When the I^2^ value is less than 50%, substantial heterogeneity will not be considered to exist in the studies. When the I^2^ value exceeds 50%, indicative statistic heterogeneity will not be considered to exist among the studies and the concealed causes of the heterogeneity will be explored. Thus, sensitive analysis or subgroup analysis will be conducted.

### 2.5 Assessment of reporting biases

Funnel plots will be used for the assessment of reporting biases and small-study effects. If 10 or more trials studies are included in the meta-analysis, a test for funnel plot asymmetry using Egger method will be conducted.^[43]^ All eligible trials will be included for funnel plots, regardless of their methodological quality.

### 2.6 Data synthesis

RevMan V.5.3 statistical software will be applied for data synthesis when a meta-analysis is allowed. The results will be expressed as RR with 95% CI for dichotomous data and WMD or SMD with 95% CI for continuous data. If no significant heterogeneity exists, the fixed-effects model will be used for data synthesis; otherwise, the random-effects model will be conducted for data synthesis. If quantitative synthesis is not appropriate such as insufficient RCTs or unidentified significant heterogeneity, we will conduct subgroup analysis or provide a systematic narrative synthesis to describe the characteristics and findings of the included trials.

### 2.7 Subgroup analysis

There is no pre-subgroup plan. If there will be adequate data, subgroups of the different chronic urticaria types and bloodletting therapy methods will be considered. If there are significant heterogeneity exists, subgroup analysis will also be applied possibly under certain circumstances.

### 2.8 Sensitivity analysis

Sensitivity analysis will be conducted to test the robustness of the primary results. Methodological quality, sample size and the effect of missing data which will be the principal decision nodes. The meta-analysis will be repeated if high significant heterogeneity exists and studies of lower quality or small sample-size will be excluded. The results will be compared and discussed according to the pooled effect size.

### 2.9 Grading the quality of evidence

The quality of evidence for all outcomes will be judged by the Grading of Recommendations Assessment, Development and Evaluation (GRADE) working group methodology. The following domains will be assessed: risk of bias, consistency, directness, precision, publication bias and additional points. The assessments will be graded into 4 levels: high, moderate, low or very low.

## 3 Discussion

Bloodletting therapy has been an important therapy during a long period in medical practice history around the world.^[19]^ At present, many trials have already reported that bloodletting therapy shows a significant curative effect for chronic urticaria. Nevertheless, no systematic reviews about BLT for chronic urticaria has published so far. This systematic review will provide a summary of the current evidence on the effectiveness and safety of Bloodletting therapy for chronic urticaria. We hope this review will facilitate clinicians when making decisions. However, this review has some potential limitations. Some relevant studies might be missed because only Chinese and English medical databases will be searched as the language barrier. Besides, different types of Bloodletting therapy therapies in included trials may cause significant heterogeneity.

## Author contributions

**Conceptualization:** Qin Yao.

**Data curation:** Xinyue Zhang, Yunnong Mu, Yajie Liu, Yu An.

**Formal analysis:** Qin Yao, Xinyue Zhang.

**Methodology:** Qin Yao, Xinyue Zhang, Yunnong Mu, Yajie Liu, Yu An, Baixiao Zhao.

**Project administration:** Qin Yao, Baixiao Zhao.

**Supervision:** Baixiao Zhao.

**Validation:** Qin Yao, Xinyue Zhang, Yunnong Mu, Yajie Liu, Yu An, Baixiao Zhao.

**Writing – original draft:** Qin Yao.

**Writing** – **review & editing:** Xinyue Zhang, Yunnong Mu, Yajie Liu, Yu An, Baixiao Zhao.
